# Multiple animal positioning system shows that socially-reared mice influence the social proximity of isolation-reared cagemates

**DOI:** 10.1038/s42003-018-0213-5

**Published:** 2018-12-11

**Authors:** Nozomi Endo, Waka Ujita, Masaya Fujiwara, Hideaki Miyauchi, Hiroyuki Mishima, Yusuke Makino, Lisa Hashimoto, Hiroshi Oyama, Manabu Makinodan, Mayumi Nishi, Chiharu Tohyama, Masaki Kakeyama

**Affiliations:** 1 Laboratory of Environmental Health Sciences, Center for Disease Biology and Integrative Medicine, Graduate School of Medicine, The University of Tokyo, Hongo, Tokyo, 113-0033 Japan; 2 Laboratory for Systems Neurosciences and Preventive Medicine, Faculty of Human Sciences, Waseda University, Tokorozawa, 359-1192 Japan; 3 Research Institute for Environmental Medical Sciences, Waseda University, Tokorozawa, 359-1192 Japan; 4 Department of Anatomy and Cell Biology, Nara Medical University, Kashihara, 634-8521 Japan; 5 Department of Clinical Information Engineering, Graduate School of Medicine, The University of Tokyo, Hongo, Tokyo 113-0033 Japan; 6 COCOSNET Ltd., 2-4-29 Kiyokawa, Fukuoka, 810-0005 Japan; 7 Laboratory for Ecological Psychology, Faculty of Human Sciences, Waseda University, Tokorozawa, 359-1192 Japan; 8 Department of Psychiatry, Nara Medical University, Kashihara, 634-8521 Japan; 9 Department of Anatomy and Embryology, Faculty of Medicine, University of Tsukuba, Tsukuba, 305-8575 Japan

**Keywords:** Social behaviour, Psychiatric disorders

## Abstract

Social relationships are a key determinant of social behaviour, and disruption of social behaviour is a major symptom of several psychiatric disorders. However, few studies have analysed social relationships among multiple individuals in a group or how social relationships within a group influence the behaviour of members with impaired socialisation. Here, we developed a video-analysis-based system, the Multiple-Animal Positioning System (MAPS), to automatically and separately analyse the social behaviour of multiple individuals in group housing. Using MAPS, we show that social isolation of male mice during adolescence leads to impaired social proximity in adulthood. The phenotype of these socially isolated mice was partially rescued by cohabitation with group-housed (socially-reared) mice, indicating that both individual behavioural traits and those of cagemates influence social proximity. Furthermore, we demonstrate that low reactive behaviour of other cagemates also influence individual social proximity in male mice.

## Abstract (web summary)

Nozomi Endo, Waka Ujita et al. present a video-based system for analysing social behaviour of groups of mice. They find that male mice reared in isolation have impaired social proximity in adulthood, but this is partially rescued by cohabitation with socially-reared mice.

## Introduction

Social behaviours are behaviours to and between other individuals or groups. Social relationships, a key determinant of social behaviour, in mammals begin after birth, first with the mother and then with other family and group members. In humans, it is well established that neglect or abuse during childhood markedly increases the risk of reactive attachment disorder^1,2^ and other major psychiatric disorders^3^. Indeed, difficulty in forming normal social relationships is an important behavioural phenotype for characterising psychiatric disorders and is often used as a diagnostic criterion^4^. In mice, numerous studies have shown that maternal separation and social isolation during development alter socio-emotional and cognitive behaviours^5–8^. However, conventional experiments on social behaviour in mice usually focus on a single individual or a single pair in a novel environment for several minutes^9,10^. Elucidating the biological basis of social behaviours developed would require a long-term behavioural analysis of multiple animals in a natural group setting.

The study of mice social behaviour has a rich tradition and has also recently been adopted by some researchers^11–19^. Importantly, these studies have demonstrated that mice social behaviours are a useful model for investigating the biological basis of social relationships. However, human observation–based approaches require tremendous amounts of work and time. Over the past two decades, computer-based systems have been developed consecutively to solve this problem^20–27^. We also developed a socially competitive task for a dozen mice^28^ using the radio frequency identification (RFID)-based system IntelliCage (TSE Systems, GmbH, Bad Homburg, Germany). We showed that mice subjected to social isolation during infancy^29^, mice with brain malformations resulting from defective neuronal migration^30^ and mice with a mutation in a *Grin1* subunit (Grin1(Rgsc174)/Grin1^+^)^31^ exhibit low competitive dominance behaviours. However, RFID-based systems also have limitations in that they can only obtain the localisation of the RFID readers that detect an RFID and do not work where no readers are placed.

Video analysis can achieve precise mouse localisation in Cartesian coordinates and provide great benefits in quantifying inter-individual distances (i.e., social proximity). Edward T. Hall suggested that the distance during social interactions depends on the relationship between the actors^32,33^. Likewise, in animal studies, social proximity regarding the number, length, and duration of interactions among animals has been used^10,34,35^. However, it remains challenging to perform these studies with multiple animals because all recently developed systems present limitations of analysis (e.g., animals must move for tracking; the analysis may be restricted to either dark or illuminated conditions; or individual identification may be impossible)^20,22,26,27^.

Herein, we develop a novel video-based behavioural analysis system for multiple small animals, which we refer to as the Multiple-Animal Positioning System (MAPS). A rodent model of social isolation has been used to investigate the effects of the rearing environment during early life. It is well established that post-weaning social isolation induces hyperactivity when an animal is tested alone in an open field^5,36^, and aggressive behaviour may be evaluated in a resident-intruder test^5,37,38^. However, the way in which social isolation affects behaviour in social conditions remains largely unknown. We employ MAPS to quantify behaviours among both socially- and isolation- reared male mice housed as cagemates. The findings of these analyses show that male mice subjected to social isolation during adolescence exhibit delayed formation of social relationships with unfamiliar male mice and that social proximity is influenced by the reactive behaviour of cagemates.

## Results

### Validation of MAPS

In MAPS, each mouse was individually identified by a mouse ID tag on its back (Supplementary Fig. 1a). MAPS then automatically acquired their individual positions based on a pattern-matching technique and saved this information to a hard disk drive together with large numbers of video images. This approach allows MAPS to perform automated long-term video tracking of each mouse under social housing conditions.

**Fig. 1:**
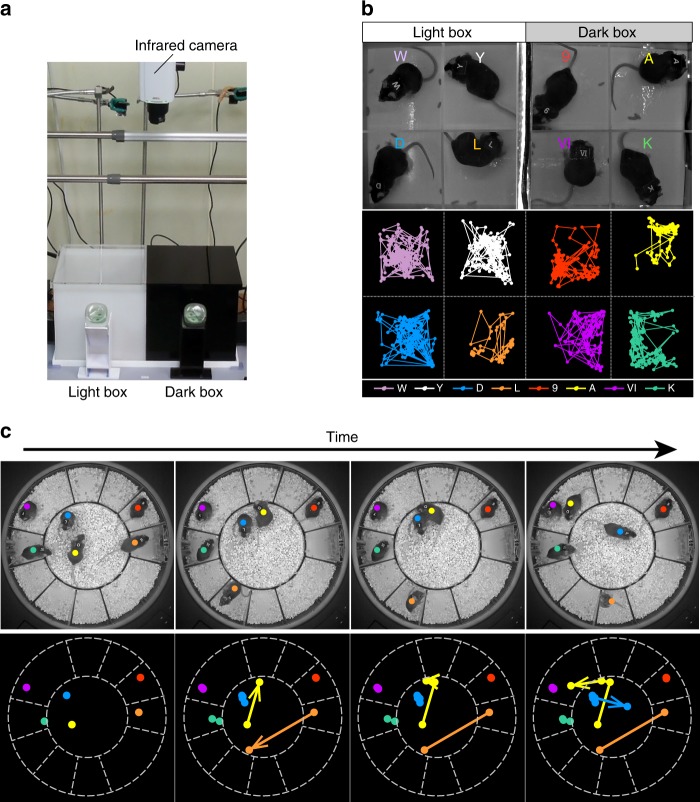
Validation of the Multi-Animal Positioning System (MAPS). MAPS is a video-and PC-based analysis system capable of determining the X–Y positions of multiple mice within a group. **a** A representative array of light and dark boxes. **b** Representative video stills recorded by MAPS (upper panel) and the corresponding tracking data (lower panel). MAPS succeeded in tracking all 8 mice without mix-up. **c** Time-lapse analysis of 6 socially housed mice in a circular chamber. Tracking data (lower panels) for the 6 mice are merged for easy viewing. MAPS succeeded in tracking all mice even when they crossed over other mice

Male mice were used in this study. Experiment 1 was performed to evaluate whether MAPS could accurately track the position of each of the eight mice recorded on video. First, each mouse was placed in one of 8 experimental chambers (4 light and 4 dark, partitioned by impassable walls; Fig. 1a), all of which were monitored by an infrared camera. The mice could move freely within their chambers but could not cross walls. The sides and top of the dark chambers were composed of acrylic that, although opaque to visible light, was able to transmit infrared light. Thus, the MAPS infrared video cameras could see into the chamber (see Supplementary Fig. 1b), and the system succeeded in determining the positions of all 8 mice, even in the dark chambers (Fig. 1b).

We next examined whether MAPS could determine individual positions when the mice were housed in the same chamber and allowed to cross paths freely. Six mice were housed together and analysed for several days (Fig. 1c). MAPS occasionally lost track of a mouse within the group when the ID was not visible in the image, especially when mice were piled on top of each other (huddled). However, MAPS could re-identify a mouse immediately after the ID reappeared. Thus, MAPS detected the positions of all six mice throughout the test, even when they crossed over other mice (Fig. 1c).

To validate the detection ability of IDs using MAPS, we compared the data from the MAPS analysis with the experimenters’ observations (in Experiment 2). Even when the mice were actively moving, MAPS could identify their IDs more than 80% of the time in both the light and dark phases (Supplementary Table 1). According to the experimenters’ observations, MAPS temporarily lost track of IDs when the tags were behind other individuals and the target mice were in an upright or recumbent position. We also confirmed, by checking the MAPS output against the manual scoring data, that the programme was effective at scoring individual locomotion distances and inter-individual distances (Supplementary Fig. 1).

### Social proximity of socially isolated mice

**Fig. 2:**
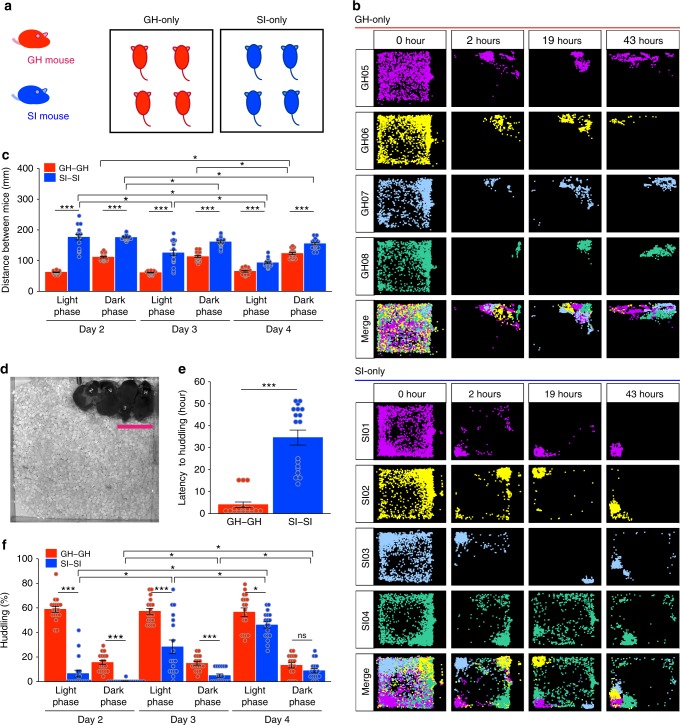
Huddling behaviour in the group-housed-only and socially isolated-only housing conditions. **a** Experimental design for the group-housed (GH) mice-only and socially isolated (SI) mice-only housing conditions. Four unrelated group-housed or socially isolated mice were housed per chamber (total *n* = 12 for each type). **b** Representative position plots over time for group-housed (upper panel) or socially isolated (lower panel) mice. **c** Inter-individual distances between group-housed pairs (GH-GH) and socially isolated pairs (SI-SI). For this index, we analysed the distance between every possible mouse pair, yielding 6 pairs in each experimental chamber (*n* = 18 total pairs in 3 chambers for each mouse type). Data are shown as mean ± SE and evaluated by three-way repeated measures ANOVA (group-housed pair vs. socially isolated pair). **d** A representative video still of huddling behaviour. Scale bar = 60 mm. **e** Latency to first huddle for group-housed (GH-GH) and socially isolated (SI-SI) pairs. Data are shown as mean ± SE and analysed by Welch two sample *t*-test (group-housed pair vs. socially isolated pair, *n* = 18 pairs each). **f** Huddling behaviour of group-housed pair mice and socially isolated pairs during light and dark phase (12-h epochs) on Days 2–4. The fraction of time spent in huddling behaviour was calculated as the percentage of 30-min bins spent huddling. See methods for more detail. Three-way repeated measures ANOVA followed by simple effects analysis for **c**, and permutation test, with one-way and with 1 between (Group) × 2 within (Day, Light/Dark) ANOVA models for **e** or **f**, respectively. *p < 0.05, **p < 0.01, ***p < 0.001

In Experiment 2, MAPS was used to examine how social experience in adolescence affects adult social proximity with unfamiliar mice. During adolescence (after weaning), male mice were either group-housed or reared in social isolation. Four adult mice that had never been co-housed were placed in one of the experimental chambers (see Supplementary Fig. 1c and 1d), and their behaviour was recorded by MAPS. The first trial in Experiment 2 was designed to detect behavioural differences, if any, between the group-housed and socially isolated mice when they interacted with other adults from the same rearing background. Thus, we examined the group-housed mice-only and the socially isolated mice-only housing conditions. Twelve mice from each group were examined (3 chambers of 4 group-housed mice or 4 socially isolated mice; Fig. 2a), and MAPS analyses were carried out for four days, starting immediately after the four mice first met in the chamber.

Plots of mouse position over time showed that both the group-housed and isolated mice began to explore the experimental chamber almost immediately (Fig. 2b, column 1). Within two hours, the group-housed mice began to stay close together in one location. Visual assessment confirmed that the mice were huddled together side by side (Fig. 2b, group-housed mice, merged). By contrast, the plots for the isolated mice revealed that during the first two hours, the mice moved to the four corners of the experimental chamber, as far away from each other as possible. The first of these isolated mice did not begin to form pairs until 10 h or more after introduction; others, however, remained separated, and it took approximately two days for all four of the isolated mice to finally huddle together in a manner resembling that of the group-housed mice (Fig. 2b, socially isolated mice, merged).

Consistent with the position distribution plots, there were differences in inter-individual distances between the isolated and group-housed mice (*p* < 0.05, Fig. 2c). After the environment became familiar (Days 2–4), inter-individual distances were greater for the isolated mice than for the group-housed mice in both the light and dark phases of the day (*p* = 8.4 × 10^-19^). Inter-individual distances between the group-housed mice were stable throughout the 4-day monitoring period and followed a circadian rhythm, with shorter distances during the light phase and longer distances during the (more active) dark phase (approximately 60 mm and 100 mm, respectively). By contrast, the inter-individual distances of the isolated mice decreased gradually over the four days (*p* < 0.05), and a clear light/dark rhythm was not observed until Day 4. Based on these results and video observations, we defined the social interaction area for each mouse as a circle with a 60-mm radius (Fig. 2d). Based on the MAPS data, approach behaviour was defined as movement into the interaction area of another individual, while huddling behaviour was defined as an average inter-individual distance < 60 mm over a 30-min period.

We found that the socially isolated mice took a longer time than the group-housed mice to begin huddling. Huddling behaviour is considered an index of social interaction when mice are at rest. To evaluate this index, we analysed huddling in two-mouse samples. Huddling began after 4.1 ± 1.2 h in the group-housed pairs (*n* = 18 pairs among 12 mice) and after 34.6 ± 3.4 h in the isolated pairs (*n* = 18 pairs among 12 mice; *p* < 0.001 using the permutation test (5000 iterations); Fig. 2e). Almost all the group-housed mice (11/12) showed huddling behaviour on Day 1, while almost all the isolated mice (10/12) failed to do so (see Supplementary Fig. 2). The group-housed mice engaged in huddling behaviour during approximately 60% of the light phase and 15% of the dark phase, indicating a clear light/dark rhythm (*p* < 0.001, using the permutation test; Fig. 2f, red bars). In the isolated mice, the total proportion of time spent huddling was lower than in the group-housed mice (*p* < 0.001, using the permutation test; Fig. 2f). Consistent with the results of the inter-individual distance analysis, the proportion of time that the isolated mice spent huddling increased gradually each day in both light and dark phases (*p* < 0.001, using the permutation test; Fig. 2f, blue bars), and these mice exhibited huddling times similar to those of group-housed mice during the dark phase on Day 4 (Fig. 2f). During the dark phase, the group-housed and socially isolated mice tended to huddle together during specific windows of time (3:00–5:00 a.m. and 5:00–7:00 a.m., respectively) (Supplementary Figs. 2 and 3).

**Fig. 3:**
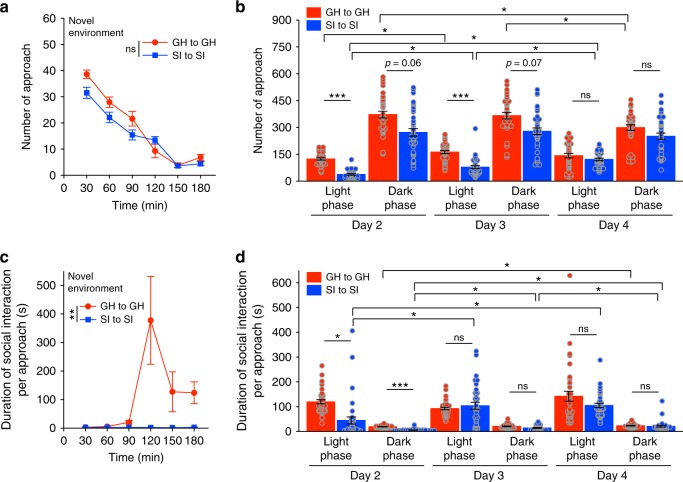
Approach behaviour in the group-housed-only and socially isolated-only housing conditions. **a**, **b** Number of approaches for group-housed (GH) and socially isolated (SI) mice in the novel environment (Day 1) (**a**) and the familiar environment (Days 2–4) (**b**). Data are shown as mean ± SE for each approach direction (group-housed mouse to group-housed mouse (GHtoGH) or socially isolated mouse to socially isolated mouse (SItoSI)). Each mouse could approach three other mice, for 36 possible approaches by the 12 mice in each condition (*n* = 36 for each approach direction). Thus, the means are the sum of all approaches for a given direction divided by 36. *p* = 0.22 in the novel environment (two-way repeated measures ANOVA) and *p < 0.05 in the familiar environment (three-way repeated measures ANOVA followed by simple effects analysis). **c**, **d** Duration of social interaction per approach for group-housed and socially isolated mice in the novel (**c**) and familiar (**d**) environments. Data are shown as mean ± SE (the average duration of each social interaction, *n* = 36 possible interaction pairs). Analysed by two-way repeated measures ANOVA in the novel environment and by three-way repeated measures ANOVA followed by simple effects analysis in the familiar environment

**Fig. 4:**
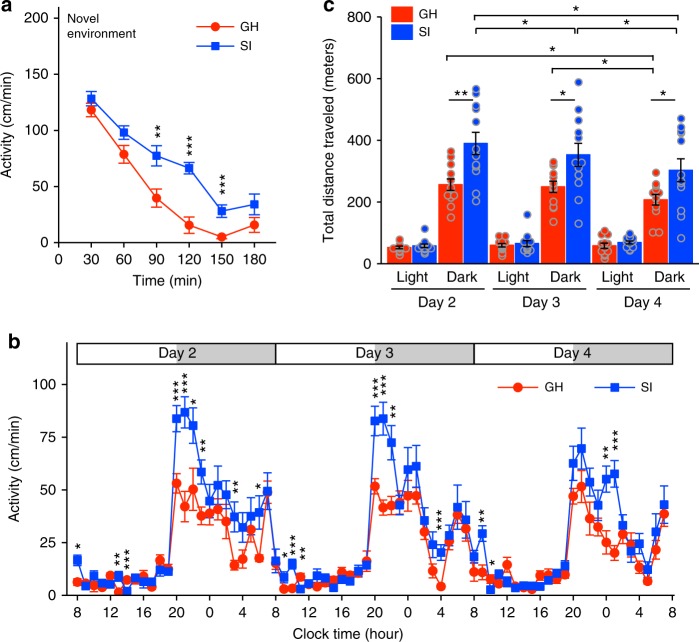
Activity in the group-housed-only and socially isolated-only housing conditions. **a**, **b** Locomotor activity of group-housed (GH) and socially isolated (SI) mice in the novel environment (Day 1) (**a**) and the familiar environment (Days 2–4) (**b**). Data are shown as mean ± SE for each mouse (*n* = 12 for each type of mouse). Two-way repeated measures ANOVA followed by a simple effects analysis. *p < 0.05, **p < 0.01, ***p < 0.001. **c** Total distance travelled in light and dark phases (12-h epochs) on Days 2–4. Three-way repeated measures ANOVA followed by a simple effects analysis. *p < 0.05, **p < 0.01

To assess social interaction when the mice were active, we compared approach behaviour immediately after placement in the experimental chamber, when the environment was novel, to approach behaviour when the environment was familiar (Days 2–4). In the novel environment, the number of approaches gradually decreased over time for both group-housed and isolated mice (Fig. 3a), which partly reflects reduced locomotor activity related to habituation (Fig. 4a). However, the isolated mice tended to approach each other less often than the group-housed mice approached each other. In the familiar environment, the isolated mice approached each other less often than the group-housed mice, especially during the light phase on Days 2 and 3 (Fig. 3b). Furthermore, the isolated mice spent less time in social interaction per approach than the group-housed mice in the novel environment (Fig. 3c), as well as on Day 2 in the familiar environment (Fig. 3d). Specifically, during periods when the mice were especially active (in the novel environment and the dark phase), the isolated mice showed a higher incidence of social interaction with durations shorter than 2 s than the group-housed mice, whereas the isolated mice showed a lower incidence of social interaction with durations over 3 s (see Supplementary Fig. 4). Thus, the isolated mice manifested reduced social interaction during both rest periods (during the light phase and after familiarisation) and active periods.

### Altered activity of socially isolated mice

Next, we analysed locomotor activity using the MAPS data. In the novel environment, the isolated mice showed higher locomotor activity than the group-housed mice (Fig. 4a), consistent with the findings of previous studies^5,36^. In addition, the isolated mice exhibited elevated locomotor activity in the familiar environment, especially at the beginning of each dark phase (Fig. 4b). The total travel distance of the isolated mice was greater than that of the group-housed mice during the dark phase in the familiar environment (Fig. 4c), indicating that social isolation in adolescence alters the activity levels of adult mice in both novel and familiar social environments. By contrast, the isolated mice still demonstrated circadian changes in activity after sufficient familiarisation with the environment.

To examine whether individual activity is affected by that of cagemates, we next assessed the correlation of the activity of mice in the same experimental chamber over 5-min periods during the light phase. Although mice spend most of their time sleeping, they occasionally wake up to eat and drink. If each mouse behaves independently, the correlations in activity between mouse pairs are expected to be weak or absent, whereas if mice influence each other’s behaviour, stronger correlations are expected. In all three of the chambers containing only the group-housed mice, high correlations were found on Days 3–4 (see Supplementary Fig. 5). In contrast, the activity of two of the isolated mice (Nos. 05 and 12) did not show any correlation with that of the other three individual mice even on Day 3; indeed, these two mice did not show huddling behaviour until Day 3 (see Supplementary Figs. 2 and 3). Thus, correlated activity between mice may reflect social relationships, and these social relationships may influence the activity of others.

### Altered social proximity by cagemates

**Fig. 5:**
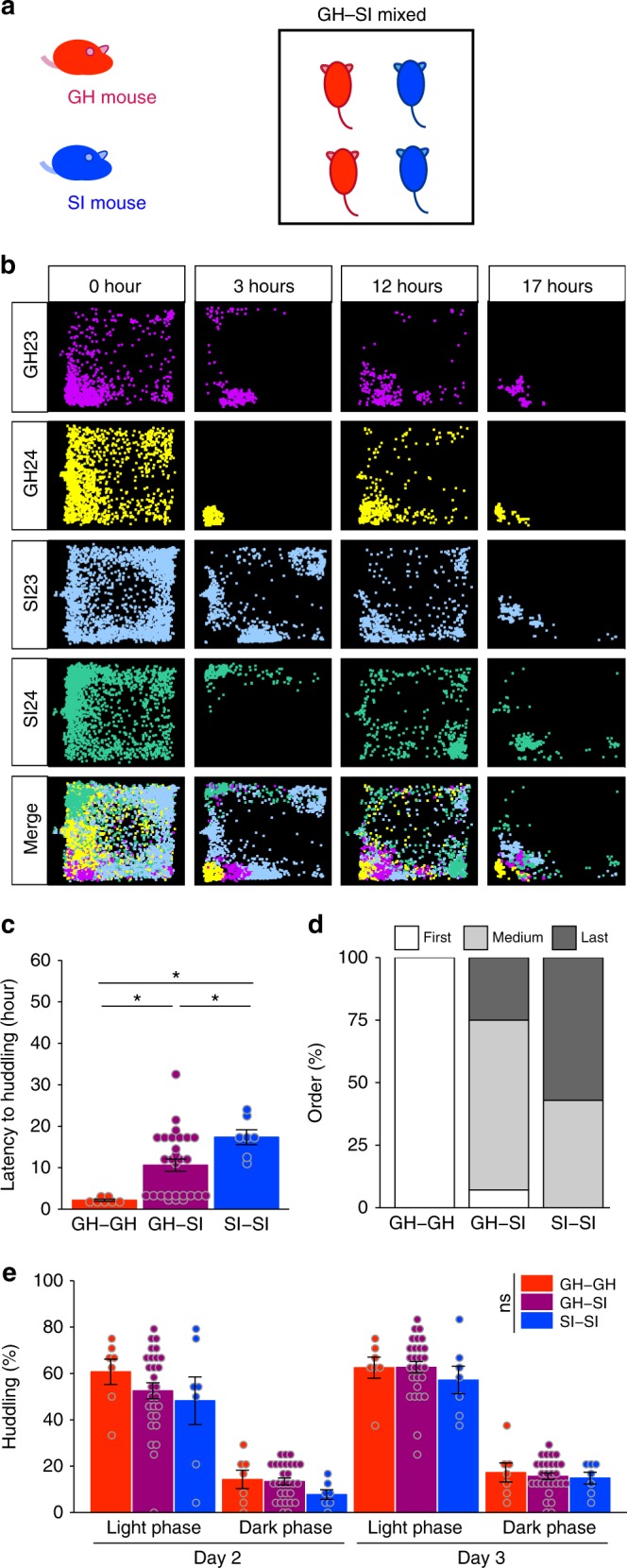
Huddling behaviour in the mixed housing condition (group-housed mice and socially isolated mice). **a** Experimental design for the mixed housing condition. Two group-housed (GH) and two socially isolated (SI) adult male mice were placed in a chamber (*n* = 14 per each type). **b** Representative position plots over time. group-housed mice (magenta and yellow), socially isolated mice (light blue and green) and merge. **c** Latency to huddling behaviour for the group-housed pair (GH-GH), heterogenous (group-housed and socially isolated) pair (GH-SI) and socially isolated pair(SI-SI). Data are shown as mean ± SE (group-housed pair; *n* = 7, group-housed and socially isolated pair; *n* = 28, socially isolated pair; *n* = 7). **d** Percentages of pairs in each huddling order category (the first, intermediate, and last to huddle) for each pair type. **e** Percent of time spent huddling for each pair during light and dark phases (12-h epochs) on Days 2–3. Permutation test, with one-way and with 1 between (Group) × 2 within (Day, Light/Dark) ANOVA models for **c** and **e**, respectively. *p < 0.05, **p < 0.01, ***p < 0.001

In Experiment 3, we examined behaviour under the mixed housing condition, in which two isolated and two group-housed mice (not examined in Experiment 2) were placed in the same experimental chamber (Fig. 5a). Position plots indicated that the two group-housed mice tended to congregate in one location for at least several hours and were only later joined by the isolated mice (Fig. 5b). The latency to huddle differed between the homogenous pair (group-housed pair and isolated pair) and heterogenous pair (group-housed and isolated pair) types (Fig. 5c; *p* < 0.01, using the permutation test (5,000 iterations)). The group-housed pair type was the fastest to exhibit huddling behaviour (latency = 2.1 ± 0.2 h; *n* = 7 pairs in 7 chambers), while the isolated pair type was slowest (latency = 17.4 ± 1.8 h; *n* = 7), and the heterogenous pairs, took an intermediate amount of time (latency = 10.6 ± 1.5 h; *n* = 28 heterogenous pairs in 7 chambers). Analysis of the huddling order within chambers showed that all 7 group-housed pairs huddled first and that 4 of the 7 isolated pairs (57.1%) huddled last (Fig. 5d), while the other 3 isolated pairs huddled before any heterogenous pair type (Fig. 5d). The difference between the homogenous pair types in terms of the fraction of time spent huddling disappeared on Days 2 and 3 (*p* = 0.30, using the permutation test (5000 iterations); Fig. 5e), which was markedly sooner than when these mice were segregated (group-housed-only and isolated-only housing conditions). The enhanced social interaction of isolated mice under mixed-housing condition suggests that the normal social behaviour of other (group-housed) mice can positively influence that of isolated mice.

**Fig. 6:**
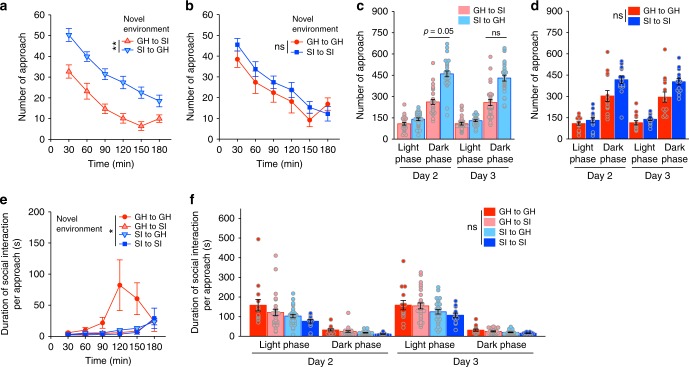
Approach behaviour in the mixed housing condition (group-housed mice and socially isolated mice). **a**–**d** Number of approaches in the novel (**a**, **b**) and familiar (**c**, **d**) environments. Data are shown as the mean ± SE for each type of approach (heterogeneous approaches: group-housed (GH) mouse to socially isolated (SI) mouse (GHtoSI) and socially isolated mouse to group-housed mouse (SItoGH); homogenous approaches: group-housed mouse to group-housed mouse (GHtoGH) and socially isolated mouse to socially isolated mouse (SItoSI)). For each mouse, there were two possible heterogeneous approaches and one possible homogenous approach. Thus, there were 28 heterogeneous approaches (left panels) and 14 homogenous approaches (right panels) possible for the 14 mice. **a**, **b** Two-way repeated measures ANOVA. **p < 0.01, GHtoSI vs. SItoGH, and *p* *=* 0.52, GHtoGH vs. SItoSI. **c**, **d** Three-way repeated measures ANOVA followed by a simple effects analysis. *p* = 0.09, GHtoSI vs. SItoGH and *p* = 0.55, GHtoGH vs. SItoSI. **e**, **f** Duration of social interaction per approach in the novel (**e**) and familiar (**f**) environments. Data are shown as mean ± SE (the mean duration of social interaction resulting from each type of approach). **e** Two-way repeated measures ANOVA. **p < 0.05. **f** Three-way repeated measures ANOVA. *p* = 0.68

We also analysed which type of mouse tended to initiate contact with the other. The number of times that isolated mice approached group-housed mice was greater than the reverse approach situation (*p* = 0.0015; Fig. 6a). No differences were found between the numbers of approaches between homogenous pair types (two-way repeated measures ANOVA; *p* = 0.52; Fig. 6b). In the dark phase on Days 2 and 3, the isolated mice tended to make more approaches than the group-housed mice (*p* = 0.09 and 0.55, respectively; Fig. 6c, d). The duration of the social interaction per approach differed among pair types, and the earlier onset of huddling behaviour by group-housed pairs was a major contributor to this difference (*p* = 0.046; Fig. 6e). On Days 2 and 3, no differences in the duration of social interaction were observed between the different pair types (*p* = 0.68; Fig. 6f), although the group-housed mice tended to exhibit longer bouts of social interaction after being approached by other group-housed mice than isolated mice exhibited after being approached by other isolated mice, with heterogenous interactions showing intermediate durations.

**Fig. 7:**
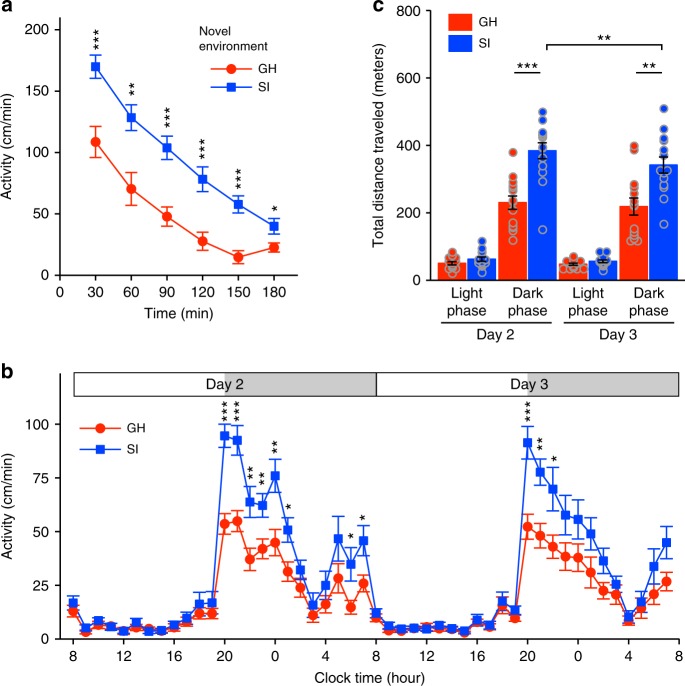
Activity in the mixed housing condition (group-housed mice and socially isolated mice). **a**, **b** Locomotor activity of group-housed (GH) and socially isolated (SI) mice in the novel (**a**) and familiar (**b**) environments. Data are shown as mean ± SE for each mouse (*n* = 14 for each type of mouse). Two-way repeated measures ANOVA followed by simple effects analysis. *p < 0.05, **p < 0.01, ***p < 0.001. **c** Total distance travelled in the light and dark phases (12-h epochs) on Days 2–3. Three-way repeated measures ANOVA followed by simple effects analysis. **p < 0.01, ***p < 0.001

The isolated mice displayed greater motor activity than the group-housed mice in both the novel environment (Fig. 7a) and the familiar environment (Fig. 7b, c) under both isolated-only and mixed housing conditions, supporting the idea that hyperactivity is a distinct trait conferred by early social deprivation and is not influenced by subsequent social environments. Under the mixed housing condition, the analysis of 5-min periods of activity revealed strong correlations even for isolated pair type on Day 2 (see Supplementary Fig. 5), similar to the observations made in the group-housed-only housing condition. Thus, the isolated mice took less time to form relationships with unfamiliar mice under mixed housing condition than in isolated-only housing, consistent with the analysis of huddling behaviour. These results indicate that it is not only individual behavioural traits but also those of surrounding individuals that can influence social proximity.

### Alteration of social proximity by the reactions of cagemates

**Fig. 8:**
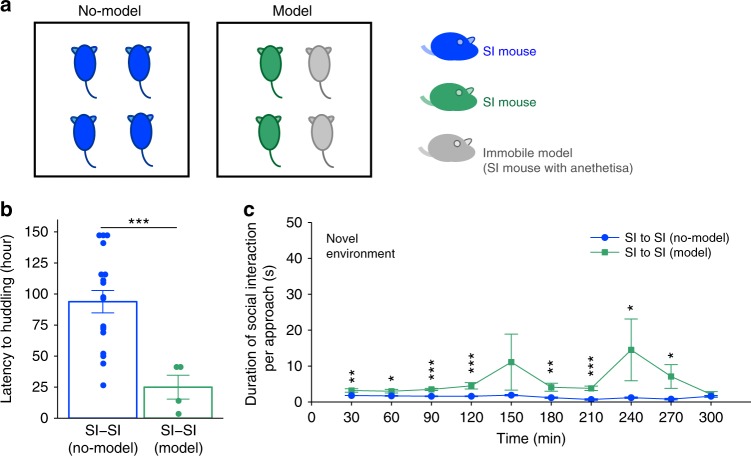
Social proximity depends on the reactive behaviour of cage mates. **a** Experimental design for the model and no-model conditions (with and without anaesthetised mice). Eight isolated mice were urethane anaesthetised as less reactive or immobile mouse model to mimic the behaviour of normal group-housed mice (immobile model). The design of no-model condition was the same as in Experiment 2. In the model condition, two non-anaesthetised socially isolated mice and two immoble model mice were placed in a chamber. Note that experimental subjects were non-anaesthetised socially isolated mice (model condition; *n* = 8, no-model condition; *n* = 12). **b** Latency to first huddling behaviour for the two conditions. Welch two sample *t*-test. ***p < 0.001. **c** Duration of social interaction per approach. Data are shown as mean ± SE. In the model condition, only one approach per mouse was possible (a non-anaesthetised mouse to another non-anaesthetised mouse); thus there were 8 approach possibilities for the 8 mice. In the no-model condition, there were 36 approach possibilities as in Experiment 2, (the average duration of each social interaction per approach for 30-min periods was used as representative of each approach direction). Two-way repeated measures ANOVA followed by simple effects analysis. *p < 0.05, **p < 0.01, ***p < 0.001

We next asked what specific traits in other mice affect the social proximity of isolated mice. In the mixed housing condition, the number of approaches made by the isolated mice was comparable to or greater than that made by the group-housed mice (Fig. 6a–d), indicating that approach behaviour may not contribute to the increased latency of the isolated mice to huddle. Visual observation of video images revealed that the group-housed mice reduced their activity or even became immobile when approached by others, suggesting that this low reactivity may be an important factor for establishing social relationships. To address this point, we studied social proximity when the recipient was less reactive, as shown in Experiment 4. To this end, we anaesthetised isolated mice with urethane as a model of less reactive (immobile) mice and compared the behaviour of non-anaesthetised isolated mice between the model condition (two non-anaesthetised isolated mice accompanied with two anaesthetised mice) and the no-model condition (four non-anaesthetised isolated mice as in Experiment 2) (Fig. 8a). Compared to the no-model condition, the isolated mice in the model condition exhibited a reduced latency to huddle (*p* = 0.00049; Fig. 8b) and longer periods of social interaction per approach (*p* = 0.0035; Fig. 8c), similar to the mixed housing condition (accompanied with group-housed mice). These results suggest that lower reactivity towards an approaching mouse contributes to the establishment of a social relationship.

## Discussion

In this study, we have developed and validated a novel video-analysis-based system, MAPS, that can identify multiple mice in social housing and specifically localise each in Cartesian coordinates over long durations in real time. MAPS analysis was conducted for 84 continuous hours (approximately 300,000 images) in this study, but this system can theoretically collect and analyse data up to the limit of the hard disk drive capacity. MAPS software is now available at http://www.waseda.jp/sem-lbn/kakeyama/MAPS.html, and the system including PC, camera and chambers are distributed by O’HARA & CO., LTD. (Tokyo, Japan).

The analysis of the behaviour of multiple co-housed mice has long been limited^11,17,20,22^. To address this limitation, an automated video analysis system was recently developed^26,27,39,40^, and combined video analysis and RFID systems for complementary use for individual identification and determination of Cartesian coordinates is also available^41–43^. In particular, the idTracker, with free access provided to the scientific community and reported for many animal species, is used to identify each individual using a set of reference images obtained from the video. However, background subtraction systems, including idTracker, possess two major limitations for mice. First, possibly because a mouse has a complex shape that includes a thin neck, an uplifted back and a long tail, the identification system can fail to recognise the correct number of mice. Second, background subtraction only functions a light field environment (Supplementary Table 2). In this context, MAPS, which performs video-based identification of IDs without calibration of the background image, provides two advantages over other systems. One advantage is a decreased burden on the identification system. The other is immediate recovery after loss of IDs, as MAPS can re-identify the ID when it becomes visible again. In other words, MAPS can identify mice immediately before and after they crossover each other. Because MAPS can impute the lost X and Y coordinates using the timepoint before mice crossover each other, there is theoretically no data loss. An additional advantage of MAPS is the ability to examine any light or dark period, even in light-dark boxes, on the basis of the infrared camera recordings and the corresponding ID tags (see Methods).

There are some limitations to the current iteration of MAPS. First, MAPS requires an ID tag on each mouse’s back, which may affect the animals’ behaviour. Therefore, we carefully considered the materials and size of the ID in an effort to minimise the stress it causes (see Methods). Second, MAPS has a lower time resolution than the other video analysis systems, and the 1 fps resolution makes it difficult to assess fine-scale behavioural interactions, such as brief aggressive or affiliative interactions. Another limitation is that the duration of monitoring is limited because the ID labels need to be replaced every 2 to 3 weeks, resulting in brief gaps in tracking when the ID number is obscured. Additionally, the number of individual animals that can be analysed (4 to 8 in the present study) is not as great as the number that can be tracked by RFID-based systems such as IntelliCage (Supplemental Table 2). It is still possible to expand the capacity of MAPS in the future to improve the time resolution for fine-scale behavioural interactions and to track a larger number of animals, and such expansion will greatly enhance the utility of the system.

To establish behavioural indices of social relationships, four adult mice (all strangers) that had been either group-housed or socially isolated during adolescence were placed in an experimental chamber, and MAPS was used to analyse their spatial distribution over time. Huddling is the primary social interaction when mice are inactive, and an approach towards another mouse is the initial step in active social interactions. The duration of social interaction can be related to both inactive and active social interaction, depending on the duration. The use of MAPS revealed that the isolated mice exhibited a longer latency to begin huddling, fewer approaches and shorter social interaction durations than group-housed mice. These results indicate that adolescent social isolation perturbs the expression of pro-social aspects of social interaction.

Mice also exhibit non-social or contingent approaches, the frequency of which depends on their general level of activity. Differentiating these approaches from true social approaches is not currently feasible in MAPS analyses. However, despite the hyperactive behaviour of the isolated mice throughout testing, these mice approached each other no more often than the group-housed mice did, suggesting that contingent approaches were infrequent relative to social approaches.

In this study, the isolated mice exhibited hyperactivity in the novel environment, consistent with other studies performed using a single mouse in a non-social environment, such as an open field^5,36^. This hyperactivity was observed in both socially isolated-only housing and the mixed housing (with group-housed mice) condition, indicating that isolation-induced hyperactivity reflects a non-social behaviour that is not affected by the behaviours of other mice. In addition, these data further confirm the ability of MAPS to detect isolation-induced hyperactivity in social housing conditions.

The circadian rhythm exerts a predominant influence on mouse activity. Mice are nocturnal, which is a non-social aspect of activity. On the other hand, the present study showed that social conditions (homogeneous or mixed housing) influenced the onset of activity. When a mouse that sleeps huddled with other mice wakes up and leaves to eat (mouse chow), other mice often follow (see Supplementary Fig. 5), which is known as a collective behaviour^11,27^. The application of MAPS analysis revealed that time-related changes in the activity of mice depend on social interaction and that such interactions can be quantitatively assessed under mixed housing condition.

The seminal observation of this study was that the isolated mice made more approaches, especially before initiating huddling behaviour, in the mixed housing condition than in the isolated-only housing condition. This behavioural feature of the isolated mice reflects both active pro-social and antisocial/unfriendly attitudes in that isolated mice are motivated to form new social relationships with others but also have a tendency to intrude into the space of others, as seen in patients with autism spectrum disorder^44,45^. These two opposing characteristics may coexist in a single mouse. In the mixed housing condition, the isolated mice also began to show huddling behaviour earlier than in the isolated-only housing condition, strongly suggesting that the behaviour of group-housed mice can influence the social behaviour of isolated mice. There are at least two possible explanations for these behavioural changes in the mixed housing condition. One is that the group-housed mice actively interact with and impel the behaviour of the isolated mice. However, the present results do not support this idea because the group-housed mice approached the isolated mice less often than the isolated mice approached the group-housed mice. Another possibility is that the behaviour(s) of the group-housed mice promotes social interaction by providing appropriate cues. This possibility is strongly supported by the relative passivity of the group-housed mice and the promotion of social interaction with anaesthetised (less-reactive/immobile) mice. The present results (Experiment 4, Fig. 8) show that decreased mobility (or immobility) in response to approach behaviour acts as a social cue that helps to hasten the onset of huddling behaviour, even in isolated mice. This observation also implies that even isolated mice can immediately recognise an associate’s behaviour and alter their own socio-emotional behaviour accordingly. There remains a possibility that the pro-social influence on isolated mice comes not from the decreased mobility (or immobility) of partners in response to an approach but from other environmental contexts. Further studies are required to assess behaviour towards a novel object rather than an anaesthetised mouse. The finding of this study that the group-housed mice also promoted huddling behaviour by the isolated mice further suggests that isolated mice spontaneously learn to huddle through experience with cagemates. Indeed, this finding is consistent with our observation that huddling behaviour in the mixed housing condition first occurred in group-housed pair type, while isolated pair type was the last to show huddling behaviour (Fig. 5c, d).

Social relationship deficits are associated with many psychiatric disorders, not only as symptoms but also as the primary cause in some cases^1–4^. MAPS will therefore be a useful tool for examining social behaviours in mouse models of psychopathology. A weak correlation between the activity of an individual and that of other cage members (as observed in socially isolated mice) may be associated with low cooperativity, a key feature associated with personality disorders^46^. We also found that some pairs of isolated mice showed latency in huddling behaviour equivalent to that of group-housed mice in the mixed housing condition. This finding raises the possibility that isolated mice are driven by pro-social motivation to form new social relationships but cannot express appropriate responses to others. This characteristic is also associated with avoidant personality disorders, social anxiety disorders, autism spectrum disorder, reactive attachment disorder and withdrawal^4^. Moreover, the fact that appropriate social interactions among others can support the formation of relationships by isolated mice suggests social interaction in heterogenous groups as a drug-free therapeutic approach for social behavioural difficulties. The analysis of mouse behaviour using MAPS may help identify cues (such as low responsivity to approach) that promote social interaction by patients with psychiatric disorders and related problems. Further studies on mouse models of psychiatric diseases are clearly warranted.

Little is known about the ability of mice to perceive their social environment and alter their own behaviour according to a situation because there are only two types of social behavioural tests focused on differences among the characteristics of other mice. One type is based on whether a mouse is familiar or unfamiliar with another mouse and includes assays such as the three-chamber test^9,47,48^, the social recognition test^49^, and the social interaction test^50,51^. The other type is based on social hierarchy and includes assays such as the tube test^52–54^ and the IntelliCage competitive task^28–30^. The present study demonstrates that mice possess the ability to alter their social behavioural traits in accordance with the social behavioural traits of other mice. A recent study using the three-chamber test showed that social isolation in adolescence induces social exploration, which is consistent with the present study^55^. Further studies focusing on this ability of mice are warranted because deficits in this ability are associated with multiple psychiatric disorders. These deficits include not only difficulties in effective communication, social participation and social relationships, as observed in autism spectrum disorder, communication disorder, personality disorder and schizophrenia but also non-social deficits, such as inflexibility and overgeneralisation, as observed in autism spectrum disorder, depression and post-traumatic stress disorder^56–59^.

In conclusion, we have (1) developed and validated MAPS for long-term behavioural tracking of multiple mice in groups; (2) shown that adolescent social isolation results in deficient social relationship formation in adulthood; (3) demonstrated that the behaviour of other mice influences the social-emotional and social proximity of socially isolated mice in mixed group housing; and (4) described how mouse studies using MAPS can assess the capacity of mice to alter their behaviour in accordance with their surroundings and in response to the social behaviours of other mice.

## Methods

### Animal experiments

The animal experiment protocols used in this study were approved by the Animal Care and Use Committees of the University of Tokyo, Waseda University and Nara Medical University. Adult male and pregnant C57BL/6 J mice were purchased from CLEA Japan (Tokyo, Japan). Male pups weaned at 4 weeks of age were used for all behavioural experiments. Mice were housed in the animal facility at 22–24 °C, with 40–60% humidity, under a 12/12 h light/dark cycle (lights on: 8:00–20:00), with free access to food and water. For convenience, the experimental day was updated at 8:00 (lights on). For Experiment 1, assessing the validity of MAPS (Fig. 1), fourteen adult male mice were used. For Experiments 2 and 3, weaned pups were divided into two equal groups. Half of these pups were socially isolated (*n* = 26), and the other half were group housed with 3 other pups from different mothers (*n* = 26). The experiments began when the mice were 13–22 weeks old. For Experiment 4, all pups weaned at 4 weeks of age were socially isolated (*n* = 28). Behavioural analyses were performed when the pups were 20 to 25 weeks old.

### ID tagging

Each mouse was tagged with a mouse ID (uniform number) on its back. The tag was attached using a Woolley rubber under pentobarbital anaesthesia, and the mouse was allowed to habituate to the ID for at least two days. According to experimenters’ observations, the mice with IDs seemed to behave normally when the anaesthetic wore off. Although no systematic analysis was performed, the results regarding hyperactivity in the novel environment and circadian changes in activity also support the notion that the application of ID tags in this study may not have affected the behavioural output and that a two-day period is enough for habituation to the ID.

The materials of the mouse ID tags were carefully determined to present high visibility under infrared camera capture. Each tag was created by printing a unique symbol on a sticker with a black background (31036, A-one 3 M Japan Group, Tokyo, Japan) on a laser printer (ApeosPort-IV C5570, Fuji Xerox Co., LTD., Tokyo, Japan). This sticker was then pasted on a retro-reflective sheet that reflects infrared light (#38150, Komatsu Process Co., Ltd. Ishikawa, Japan). The printed symbol was also pasted on a plastic pedestal (1.5 cm × 1.5 cm). Mice with IDs were placed in the experimental chambers, and their behaviours were video recorded and analysed using MAPS as described below.

### MAPS

MAPS is a video- and PC-based analysis system capable of determining the position of each mouse under social housing conditions (O’HARA & CO., LTD., Tokyo, Japan) (MAPS software is available at http://www.waseda.jp/sem-lbn/kakeyama/MAPS.html). Video images of mice in the experimental chamber were recorded (1024 × 768 pixels or 1280 × 960 pixels) using a network video camera (P1344 or P3364-V 6 mm, AXIS Communications, Lund, Sweden) under infrared illumination. MAPS can record high-resolution video images in a network-attached storage (NAS) hard disk drive, manage the image database, and perform automated pattern-matching-based ID identification, specification of ID (animal) orientation, and long-term video tracking of each individual mouse in social housing conditions.

The tops of all experimental chambers were composed of acrylic plates capable of high infrared light transmission. MAPS collects infrared images under infrared illumination; therefore, all experimental areas were accessible to MAPS, even in the dark and when covered by visually opaque walls. Mice were housed in these chambers throughout the experiment, and all video images (1 fps) were recorded by MAPS.

Conventional tracking systems for multiple individuals are based principally on shape recognition (detection of animal shapes from images) and time series analysis. However, if the system fails to track the animal’s identification information, it is difficult to automatically re-identify a given animal, and experimenters must manually recover the systematic error. In experiments using MAPS, a mouse ID occasionally disappears from the image during the course of long-term social housing (e.g., when mice overlap or their bodies are tilted). However, unlike conventional tracking systems, MAPS can identify mouse IDs in every image, where visible, and re-identify an ID immediately. In the present study, when MAPS lost a mouse ID, the lost X and Y coordinates were imputed using previous data from when the ID was last identified.

### Validation of MAPS (Experiment 1)

Eight mice were housed in two chambers (20 cm × 20 cm × 20 cm): one light and the other dark (Fig. 1a). The light box consisted of visually white translucent walls and a visibly transparent top, and the dark box had black walls and a black top. In the first experiment, we divided each chamber into 4 smaller chambers (Fig. 1b), with a mouse placed in each. The mice were allowed to move freely within each smaller chamber. We then recorded a 10-min infrared video that covered all 8 of the small chambers (all 8 mice).

In the next experiment, we used a circular box (43 cm in diameter, Fig. 1c) in which a circular centre field was surrounded by 10 small chamber fields. The mice could move freely between the centre field and each of the chamber fields. Six mice were placed in a chamber together, and an infrared video of the entire box was recorded over several days. MAPS recorded all video images at 1 fps and determined the position of each individual (i.e., identified the mouse and determined its X and Y coordinates) continuously throughout the test.

The analysis of positioning data from MAPS and generation of tracking images were performed using R version 3.3.1, a free software environment for statistical computing and graphics^60^.

### Group-housed-only and socially isolated-only housing conditions (Experiment 2)

A total of 12 group-housed and 12 socially isolated adult male mice were used. Four group-housed or socially isolated mice with no previous contact were placed in separate group-housed-only and socially isolated-only housing chambers (both 24 cm × 24 cm × 20 cm), with visually black walls and a visibly transparent top, accompanied by a food area (7.8 cm × 3.0 cm × 3.0 cm) (Fig. 2a and Supplementary Fig. 1c and 1d), from 14:00 to 17:00 on Day 1. Video recordings of the experimental chambers were obtained under infrared illumination, and mouse behaviour was examined using MAPS over four days.

### Mixed housing condition (Experiment 3)

The group-housed and socially isolated adult male mice that had not been used in other experiments were placed in mixed groups consisting of 2 group-housed and 2 socially isolated mice per chamber (Fig. 4a), and mouse behaviour was examined using MAPS for three days.

### Anaesthetised mice as a model (Experiment 4)

Twenty-eight socially isolated mice that had not been used in other experiments were employed in this experiment. To mimic group-housed mice with less reactive behaviour, eight socially isolated mice were anaesthetised with urethane (1.3 g/kg of body weight, i.p.) immediately before the behavioural test. Two socially isolated mice (experimental subjects) and two anaesthetised socially isolated mice were placed in each of four experimental chambers (model housing condition). As a control, four socially isolated mice (experimental subjects) were placed in each of three chambers as Experiment 2 (no-model housing condition). Mouse behaviour was examined using MAPS until huddling behaviour was observed by an experimenter. Because anaesthetised socially isolated mice were used as a social environmental factor, we analysed only the behaviours of non-anaesthetised socially isolated mice in this experiment.

### Data analysis

Distribution plots: To visualise the changes in mouse position over time, we constructed cumulative distribution plots for each hour.

Distance between mice: Inter-individual distances between all mouse pairs (in mm) were calculated once per second. In addition, we divided each day into 30-min periods or 12-h periods (light or dark phase) and calculated the mean inter-individual distance (mm/seconds) for each period.

Approach behaviour: The social interaction area was defined for each mouse as a circular area with a 60-mm radius surrounding the ID, which was the maximum distance over which two mice could physically reach each other (Fig. 2d). Approach behaviour was defined as the movement of a mouse into the interaction area of another individual. The onset of approach behaviour was defined as the time when the inter-individual distance between two mice became less than 60 mm. The approaching and approached individuals were defined as the mice that had travelled the longer and shorter distances, respectively, within the 3 s before the onset of approach behaviour. The mean number of approach behaviours was calculated for 30-min periods in the novel environment or for 12-h periods in the familiar environment.

Duration of social interaction: The duration of social interaction was defined as the time from the onset of approach behaviour until the inter-individual distance became greater than 60 mm. The mean duration per approach (seconds) was calculated for 30-min periods in the novel environment or 12-h periods in the familiar environment.

Huddling behaviour: Huddling behaviour was defined as a mean inter-individual distance of less than 60 mm during a 30-min period. Under social housing conditions, two or more mice often sleep together. Even when a mouse wakes up and patrols, the huddling behaviour occasionally continues when the mouse returns. To measure this huddling behaviour, we divided each day into 30-min periods and calculated the mean inter-individual distances between all pairs of mice for each period. The percentage of huddling behaviour was calculated as 100 × the number of huddles/24 (the number of 30-min bins in the light or dark phase). The latency to huddling behaviour was defined as the 30-min bin during which the first huddle occurred. Note that huddling behaviour involves two individuals, and the experimental subject is therefore actually a pair of mice (group-housed pair, socially isolated pair, or heterogenous (group-housed and socially isolated) pair) in this case, rather than an individual mouse. There were six possible pairwise relationships that could be formed by four animals in one experimental chamber. In each chamber, we ranked the order in which each pair first huddled. For each pair type, we then calculated the percentages of pairs that fell into the first (earliest) rank, middle ranks and last rank.

Activity: The locomotor activity of each mouse was calculated as the distance travelled per second (mm/s). The mean activity (mm/s) was calculated for each period and then converted to cm/min. The correlation of activity between each mouse pair was calculated using the mean activity of each mouse over a 5-min period during the light phase.

### Statistical analysis

The sample sizes in Experiments 2, 3, and 4 were determined on the basis of our pilot experiments and on the number of mice required to detect a 20% change in behavioural indices. There were no clear differences between chambers in any experiment (data are available at figshare with the identifier DOI^61^. The data were analysed by Welch’s two sample *t*-test (Figs. 2e and 8b); two-way repeated measures ANOVA (Figs. 3a, c, 4a, b, 6a, b, e, 7a, b and 8c); and three-way repeated measures ANOVA (Figs. 2c, 3b, d, 4c, 6c, d, 6f and 7c). Multiple comparisons between two or more groups were conducted using Holm’s sequentially rejective Bonferroni procedure. When a significant interaction between factors was observed by ANOVA, the simple main effect test was performed for the mouse type × action or the mouse type × light/dark phase × day interaction. Permutation tests (5,000 iterations) with a one-way ANOVA model (Figs. 2e and 5c) or with a 1 between (Group) × 2 within (Day, light/dark phase) ANOVA model (Figs. 2f and 5e) were also performed. Data processing and statistical analyses were conducted using R version 3.3.1^60^ and anovakun (version 4.8.0)^62^. Statistical significance was set at *p* < 0.05 (two-tailed) for all tests. Graphs were generated using R and the ggplot2 package^63^.

### Code availability

The applied code is available at figshare with the identifier 10.6084/m9.figshare.7016795^61^.

## Electronic supplementary material

Supplementary Information

Supplementary Data 1

Supplementary Data 2

Supplementary Data 3

Description of Additional Supplementary Files

## Data Availability

The data supporting the findings of this study are available at figshare with the identifier 10.6084/m9.figshare.7016795^61^. The data source underling the graphs in the main figures is available in Supplementary Data 1 (Figs. 2, 3, and 4), 2 (Figs. 5, 6, and 7), and 3 (Fig.8).
