# Supplementary material for: Multiple animal positioning system shows that socially-reared mice influence the social proximity of isolation-reared cagemates
Source: Commun Biol. 2018 Dec 11;1:225. doi: 10.1038/s42003-018-0213-5 (PMC6290015; doi:10.1038/s42003-018-0213-5)
Supplement: Supplementary file 1 — Supplementary Information [file 42003_2018_213_MOESM1_ESM.pdf]

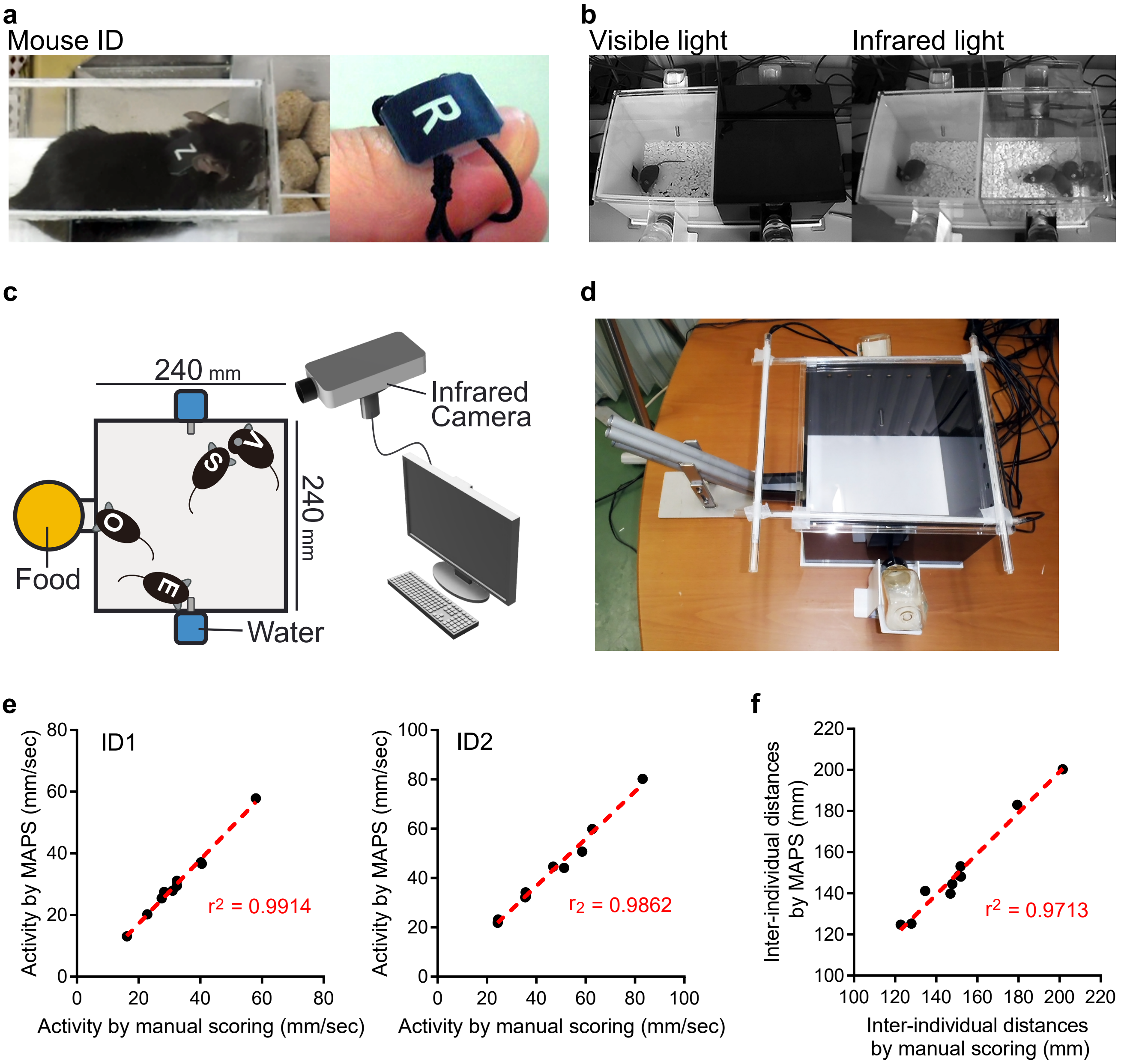


## Supplementary Figure 1

**(a)** Representative photographs of mouse ID. **(b)** Light and dark boxes taken by visible (left) and infrared light (right). **(c)** Overview and **(d)** photograph of the experimental chamber used in Experiment 2, 3 and 4. **(e, f)** The result of comparing a video analysis data by maps and the experimenter`s manual scoring. The X-Y coordinate of two free-moving mice in a chamber were obtained for 10 minutes, and calculated in locomotor activity **(e)** for ID1(left) and ID2 (right), and inter-individual distance **(f)** as the mean of 1-min bins.

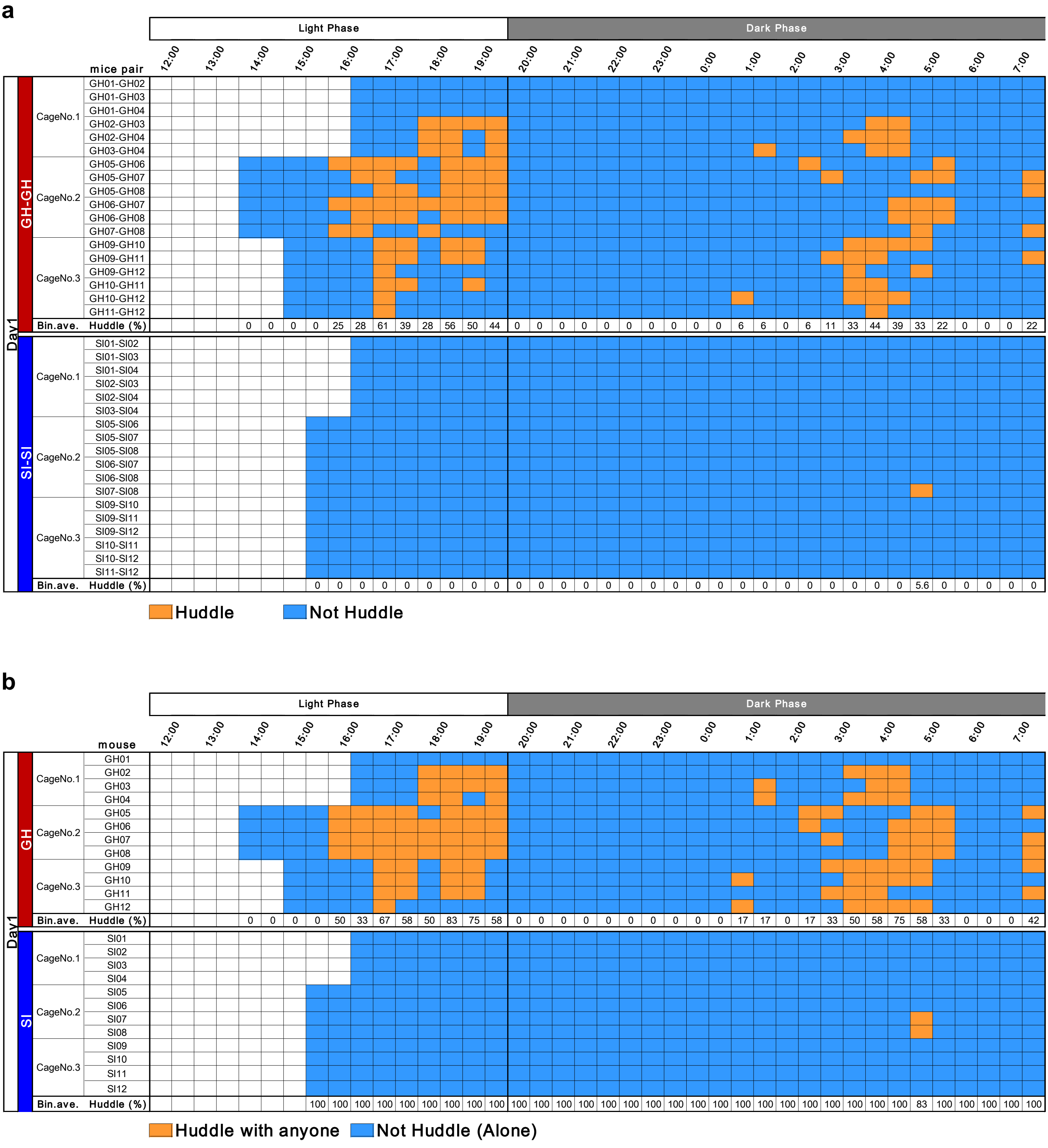


## Supplementary Figure 2

Heat maps of "huddling behaviour" status on the Day1 (30 min-bin) in the GH- and SI-only housing condition. **(a)** A heat map showing "huddling behaviour" status of each mice pair. An orange cell; the 30 min-bin that the mice pair exhibited "huddling behaviour" , and a blue cell; the 30 min-bin that the mice pair did not exhibited "huddling behaviour" . **(b)** A heat map showing huddling with anyone, or alone for each mouse. An orange cell; the 30 min-bin that the mouse exhibited "huddling behaviour" with anyone, and a blue cell; the 30 min-bin that the mouse did not exhibited "huddling behaviour" (= alone).

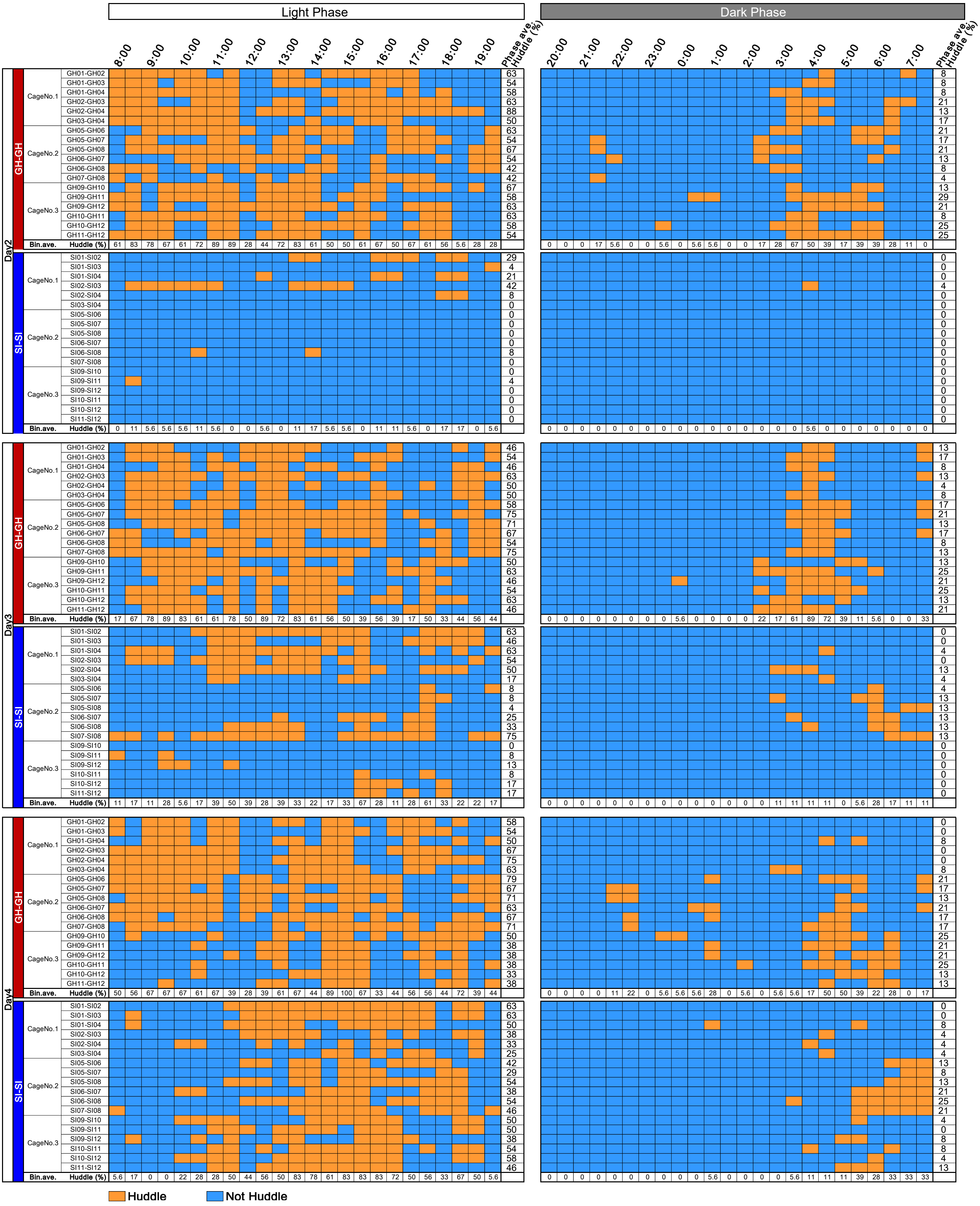


## Supplementary Figure 3

A heat map showing "huddling behaviour" status of each mice pair on the Day 2-4 in the GH- and SI-only housing condition. An orange cell; the 30 min-bin that the mice pair exhibited "huddling behaviour" , and a blue cell; the 30 min-bin that the mice pair did not exhibited "huddling behaviour" .

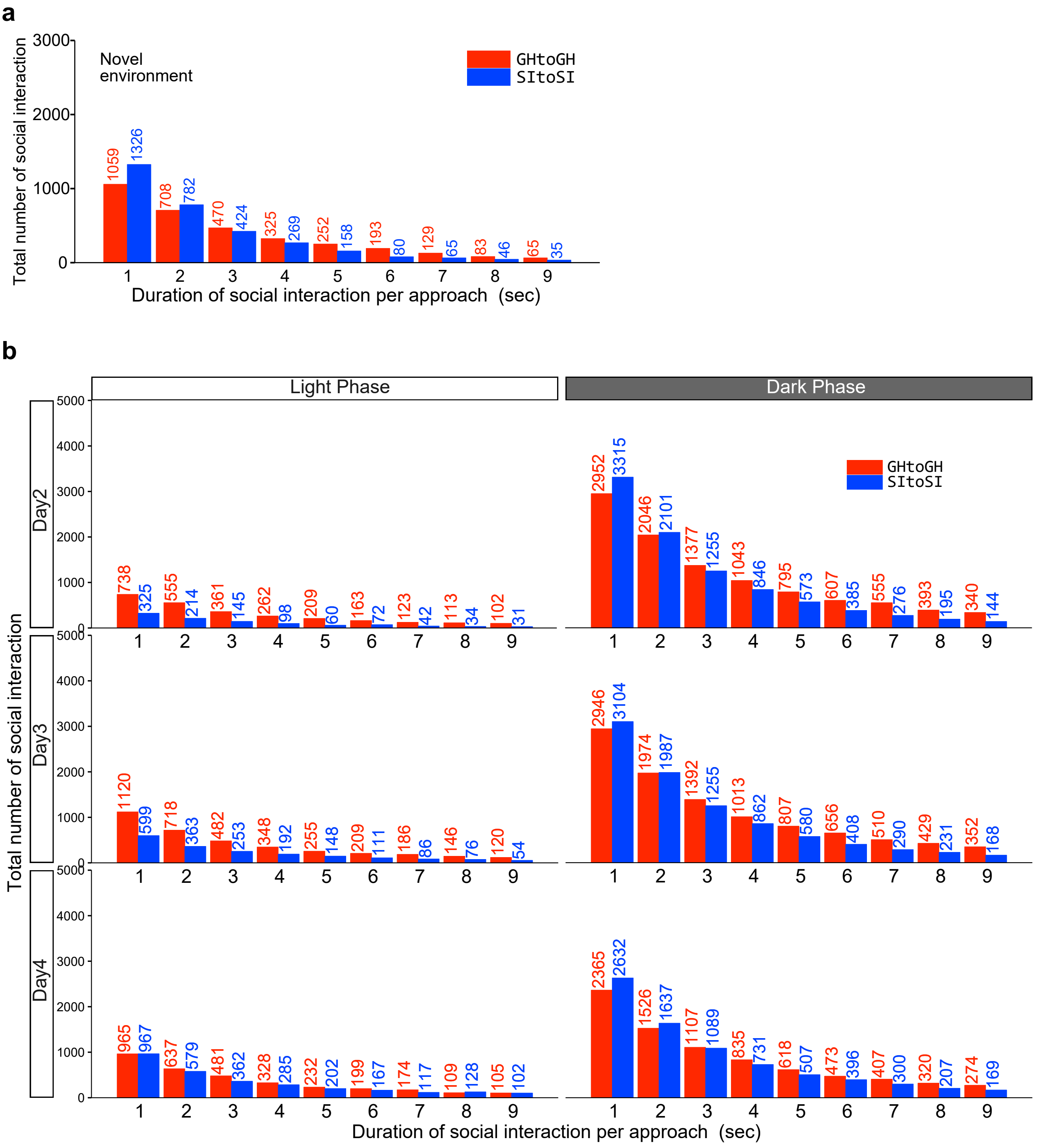


**Supplementary Figure 4**

Duration histograms of social interaction (<10 sec) in the **(a)** novel and **(b)** familiar environments in the GH- and SI-only housing condition. Total number of social interaction of each type (n = 36 for each approach direction) for each duration (1 sec-bin) was shown.

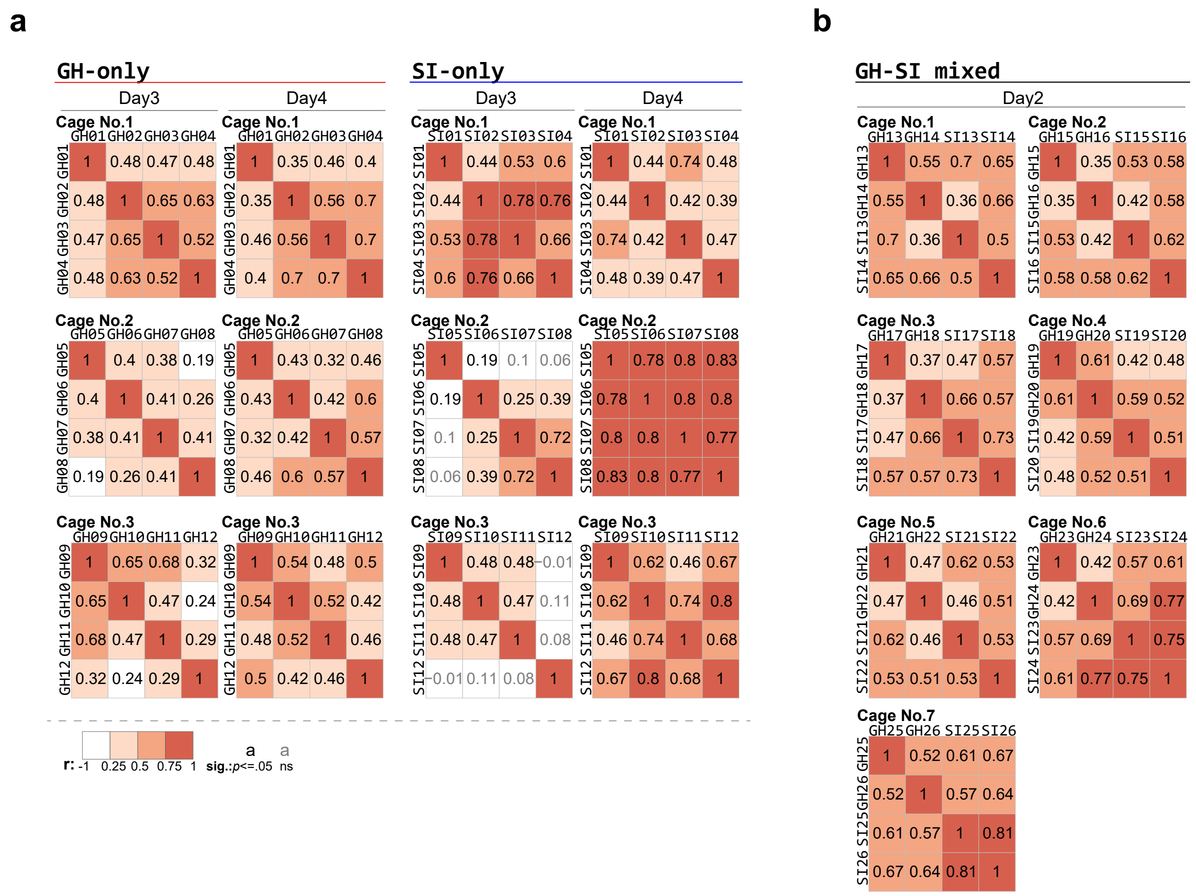


## Supplementary Figure 5

Heat maps showing representative correlation matrices of the correlations of 5-min periods of activity among mice in the same experimental chamber during the light phase on Days 3 and 4 in the GH-only and SI-only housing conditions **(a)** and on Day 2 between mice in the GH−SI mixed condition**(b)**. The IDs of GH and SI mice are shown as GH 01 to GH 26 and SI 01 to SI 26, respectively. Black colour type indicates p ≤ 0.05, grey colour type indicates ns.

**Supplementary Table 1.** Percentages of successfully detected frames by MAPS automated analysis.

| Group | number of IDs | Light Phase | Dark Phase |
|---|---|---|---|
| SI | 8 | 79. 2 ± 4.2 % | 81.4 ± 5.1 % |
| GH | 8 | 82.3 ± 4.9 % | 80.9 ± 3.8 % |

*mean ± S.E. (n=8 in each group)

*Data were obtained from Experiment 2 (four mice in a chamber in the GH-only and SI-only housing condition) by MAPS automated analysis and confirmed by experimenters' observation.

*Successfully detected frames were calculated from 60 min. extraction images when mice were relatively active.

**Supplementary Table 2.** Summary of publications using automated or video-based home cage analysis in group-housed mice.

| Reference | Product | Individual identification | Advantages | Disadvantages |
|---|---|---|---|---|
| Arakawa et al., (2007)<br>Pobbe et al., (2010) | Visible Burrow system. (VBS) | Manual observation of video images | | Requires long-term observation and analysis by expert observers. |
| Galsworthy et al., (2005)<br>Endo et al., (2012)<br>Benner et al., (2014)<br>Ishii et al., (2015)<br>Ujita et al., (2018) | IntelliCage | RFID | Extremely precise and automated identification | Identification restricted in 4 places (entrance of corner chambers). |
| Bains et al., (2016) | Home Cage Analysis (HCA) system | RFID combined with manual observation of video images | Same as the above.<br><br>In manual observation, objectivity can be guaranteed by video record. | RFID identification restricted to where the reader allocated.<br><br>In manual observations, long-term observation and analysis by expert observers are required. |
| Alexandrov et al., (2015) | PhenoCube system (video analysis combined IntelliCage) | Video analysis combined IntelliCage | Same as IntelliCage in RFID analysis<br><br>In video analysis, locomotion can be quantified as the total value for all mice in a field. | Same as IntelliCage in RFID analysis<br><br>Lack of individual data in video analysis<br>Animals must move.<br>Not applicable for heterogeneous housing |
| Pérez-Escudero et al., (2014) | idTracker | Video analysis (background subtraction) (2 or 4 mice) | High-frame-rate tracking analysis<br>Does not require marking.<br>Excellent achievements, especially in fish studies | Subtraction works only in light conditions.<br>Requires setting up background subtraction parameters. |
| Endo et al., 2018)<br>(present study) | MAPS | Video analysis (pattern matching of markers) | Identification and tracking of up to 6 mice | Low frame rate<br>Requires marking |
