# Supplementary material for: Multiple animal positioning system shows that socially-reared mice influence the social proximity of isolation-reared cagemates
Source: Commun Biol. 2018 Dec 11;1:225. doi: 10.1038/s42003-018-0213-5 (PMC6290015; doi:10.1038/s42003-018-0213-5)
Supplement: Supplementary file 5 — Description of Additional Supplementary Files [file 42003_2018_213_MOESM5_ESM.docx]

**Description of Additional Supplementary Files**

**File Name**: Supplementary Data 1

**Description**: Source data of Figs. 2, 3 and 4.

**File Name**: Supplementary Data 2

**Description**: Source data of Figs. 5, 6 and 7.

**File Name**: Supplementary Data 3

**Description**: Source data of Fig. 8.
